# Incidental findings from clinical sequencing in Greece: reporting experts’ attitudes

**DOI:** 10.1007/s12687-014-0194-0

**Published:** 2014-07-22

**Authors:** E. G. Gourna, N. Armstrong, S. E. Wallace

**Affiliations:** Department of Health Sciences, University of Leicester, Adrian Building, Leicester, LE1 7RH UK

**Keywords:** Incidental findings, Experts’ attitude, Clinical sequencing, Interviews, Greece

## Abstract

Unprecedented progress in sequencing technologies and decreasing cost have brought genomic testing into the clinical setting. At the same time, the debate in the literature concerning the return of incidental findings (IFs) has made this an important issue internationally. These developments reflect a shift in genetics that will also affect smaller countries, such as Greece, that are just starting to implement these technologies and may look to other countries for examples of good practice. Ten in-depth interviews were conducted with Greek experts in clinical sequencing. Previous experiences and attitudes toward IFs and clinical sequencing were investigated as well as views on the existing policy regarding managing genetic information generated through testing. . Interviews were analysed using thematic analysis. All participants reported the lack of any legal or other supportive mechanism. IFs are currently managed at a “local” level, i.e. within the clinic or the laboratory in an ad hoc way. All participants thought that clinically valid and actionable IFs should be returned, but always with caution and in respect to patients’ wishes, although several experts reported returning IFs according to their clinical discretion. Experts reported that most patients ask for all tests available but they felt that more counselling is needed to understand and manage genetic information. Due to the lack of any supporting mechanisms, professionals in Greece, even those with established experience in the field of genetic and genomic testing, have difficulties dealing with IFs. All experts agreed that it is now time, before the full integration of genomic testing into everyday clinical practice, for guidance to help Greek physicians work with patients and their families when IFs are discovered.

## Introduction

The development of whole-exome and whole-genome technologies (next generation sequencing (NGS)) has been revolutionary, and their use as a diagnostic tool in clinical sequencing has transformed everyday clinical practice. With costs expected to fall to  $1,000 per genome (Check Hayden 2014) and the continuing development of software to facilitate data interpretation, the integration of NGS into the clinical setting (Lyon et  al. 2011) is moving very quickly. This means there has been limited time available for public dialogue regarding its potential implications. One of the main issues coming out of the use of NGS is the increased possibility of discovering incidental findings. Incidental findings (IFs) have been defined as findings with potential health or reproductive importance to individuals discovered during diagnostic testing or during research but falling outside the diagnostic indication for which the test was ordered (Wolf et  al. 2008). A recent publication (March 2014) from the Medical Research Council (MRC) and the Wellcome Trust in the UK provides a clearer framework about IFs from research settings (MRC and Wellcome Trust 2014) and reflects the ongoing effort to provide clear guidance.

IFs in the clinical setting first appeared in relation to imaging tests (Morris et  al. 2009; Lumbreras et  al. 2010), and the phenomenon quickly spread into genetic and genomic testings. Until recently, little guidance was available regarding how IFs from clinical genomic testing are to be dealt with. Available recommendations concern mainly return of IFs from research (Cassa et  al. 2012) and have been criticised as inconclusive (Zawati and Knoppers 2012; Knoppers et  al. 2013; Lawrenz and Sobotka 2008).

The most specific guidelines about IFs from the clinical setting currently available are from the American College of Medical Genetics and Genomics (ACMG) published in 2013 (Green et  al. 2013). ACMG recommends that when conducting clinical sequencing, regardless of the diagnostic indication for which the test is being conducted, or the age of the patient, laboratories should actively look for and report mutations on listed genes. The variants included in the list were medically actionable and concerned conditions with well-established genetic aetiology. Although these recommendations were revised on April 2014 (ACMG 2014) allowing patients to opt out from receiving IFs, they still represent the beginning of a discussion that has dominated the literature for the last 15 months.

Additional guidance comes from the Presidential Commission for the Study of Bioethics Issues (USA). In their report published in December 2013, they recommended that regardless the setting “practitioners should inform potential recipients about the possibility of incidental findings” and ascertain recipients’ intentions about receiving them ahead of time (BioethicsGov 2013). At a European level, the European Society of Human Genetics in their “Call for Prudence” encourage the use of targeted tests to avoid IFs, while acknowledging that “patient’s right not to know may sometimes have to be secondary to clinical geneticists’ professional responsibilities” (van El et  al. 2013a, b).

These recommendations and the discussion surrounding ACMG recommendations (Green et al. 2013; Couzin-Frankel 2013; Klitzman et  al. 2013; McGuire et  al. 2013; Bombard et  al. 2013; Ross et  al. 2013) and their early adoption (GenomeWeb 2013; Heger 2013) highlight the fact that this field is moving very quickly and brings to the surface fundamental differences in ethical views. Experts from the USA and Europe have expressed their reservations about the implementation of the ACMG recommendations suggesting that more evidence is needed and that these recommendations might not be appropriate for all types of clinical sequencing (Middleton et  al. 2014; Burke et  al. 2013; Hickner 2013).

These guidelines could seem attractive for adoption by smaller counties where there are currently no guidelines and where resources are limited to produce guidelines by themselves, such as in the case of Greece. However, to ensure what guidelines are appropriate for each country, various stakeholders need to be approached. Given the controversy, it is crucial to ascertain the attitudes of different stakeholders. These stakeholders are likely to include, among others, professionals and experts in genomics, patients, and the lay public. Input from different countries should also be sought to compare and contrast different attitudes. These perspectives could then be used to support the creation of guidelines in other countries that would better reflect cultural differences.

Our aim in this paper is to describe the existing situation in Greece and report the first study investigating Greek experts’ attitudes toward return of IFs from clinical sequencing. We believe that the input from Greece could add to the broad literature and encourage an international dialogue between countries with strong traditions in governance of genetic testing and other countries, such as Greece, that are just beginning to apply these technologies and are looking to other countries for examples of public health policies.

## The Greek context

Currently in Greece, patients have access to genetic testing through both the public and the private sectors. An individual with a diagnostic indication or family history for a genetic condition can consult a physician who will refer the individual to a specialised clinic or one of the available genetic laboratories. Most of the public laboratories are linked to a university hospital. Such laboratories can be found in some of the major cities in Greece, such as Athens, Thessaloniki, Patra, and Ioannina. In the public sector, it is currently unclear which, if any, of the costs will be covered by health insurance.

Alternatively, an individual can go directly to one of many private laboratories, located in most cities in Greece, and ask for any available genetic test (Intergenetics 2014). The cost of the test will need to be paid by the individual unless he or she has private insurance willing to cover some of the expenses.

In 2013, the Hellenic Association of Medical Genetics (HAMG) and the Hellenic Society of Medical Genetics (HSMG 2011), the two professional association of its type in Greece, had 240 registered members. These included clinicians, dentists, biologists, and biochemists working in genetics (HAMG 2013: content in Greek). No genetics-related medical specialty is recognised by the state. More specifically, neither the specialty of clinical geneticist nor the specialty of lab-based geneticist is recognised. Professionals working in genetic and genomic testing have gained their expertise either abroad, where such specialist training is available, or through working in this area for many years. There is also no specialist training for or a recognised speciality of genetic counselling. This role is taken on by clinicians and geneticists who provide this service as a part of their clinical relationship with their patient.

Genetic testing in Greece is regulated by the legal framework that applies to health services as a whole. The ability of users to access genetic services is regulated to protect patient rights. According to law number 2472/1997 concerning the use of personal data (Greek Government 1997), all health-related data are considered “sensitive” and can therefore be collected, stored, or processed only by the Hellenic Data Protection Authority and only after the individual’s informed consent. An exception can be made if the processing concerns health data and is conducted by a person who is, by training, working in health services and is bound by confidentiality and deontological codes of practice. The processing must also be necessary for clinical services, such as medical prevention, diagnosis, nursing, or the management of health services.

Any institutional guidance on sharing personal data between doctors and their patients reflects international codes of practice such as UNESCO’s Universal Declaration on the Human Genome and Human Rights (1997) (UNESCO 1997) and International Declaration on Human Genetic Data (2003) (UNESCO 2003). These declarations seek to provide guidance for best practice in the protection of patient data deriving from genetic tests. Additionally, the Oviedo convention, which only addresses the return of findings from research, is integrated into Greek legislation with law number 2619/1998 (Greek Government 1998), and states that “everyone is entitled to know any information collected about his or her health. However, the wishes of individuals not to be informed shall also be respected”.

One of the reasons there is no guidance for clinicians in Greece is because there are no organisations formally responsible for the creation of good practice guidelines. Clinicians rely on the law concerning Medical Ethics (number 3418/2005) (Greek Government 2005) for general guidance regarding their duties toward patients and their families. According to this law, physicians are responsible for developing a relationship of mutual trust with their patient and respecting his or her wishes and beliefs. The physician bears a “duty of truth” toward the patient. The patient should be fully and comprehensibly informed and should have understood the risks of the test. The physician shall respect an individual’s wish not to be informed. In this case, the patient has the right to ask the physician to exclusively inform another or other people of the patients about their condition and the results of medical investigations. The physician shall not disclose confidential information to anyone unless the patient has requested otherwise.

There is a need for more specific guidance regarding genetic testing and return of results. This issue is important and will become more so with the increasing integration of genetic testing into clinical practice and the use of less targeted genetic testing that might produce more results of unknown significance. It remains unclear what form this guidance could best take; it may be in the form of a law or a set of guidelines or recommendations by a professional organisation, which could be sufficient for the transitional period until genomic testing is fully integrated in the clinical setting.

Our goal is to investigate experts’ attitudes toward clinical sequencing and return of IFs in order to help us gain a better understanding of the current situation in Greece.

## Methods

Ten in-depth interviews were conducted with Greek experts acting as key informants. We have defined experts as clinicians, geneticists and professionals with a bioethical background with experience of clinical sequencing. The number of professionals in Greece with extensive experience in genomic testing is limited. However, the sample, while not typical of the general population, is considered as typical of Greek experts in genomic testing.

Given that there are no official records of genetic/genomic professionals in Greece, professionals were invited according to their experience, as evidenced through their published work on genomic testing and conference presentations in Greece. There have been no publications about IFs in clinical sequencing in Greece or about the issue in the Greek language. Four experts were initially identified, and additional professionals were recruited using a snowballing technique (Wimmer and Dominick 2011).

In total, 20 experts working with genetic and genomic testings in either the public or the private sector were invited to participate via email. Fifteen experts responded, of whom five did not regard themselves as sufficiently experienced or currently working in a relevant area. The remaining ten agreed to be interviewed and an email was sent to arrange a meeting at a time and place of their convenience. All participants received an information leaflet and signed a consent form at the beginning of their interview. Interviews were performed in interviewees’ preferred language. All interviews were conducted by EGG. This study was approved by the University of Leicester College of Medicine and Biological Sciences Ethics Committee.

A draft topic guide was used to facilitate discussion and ensure that all topics of interest were covered. In addition to this topic guide, a vignette, describing a scenario where an IF is discovered in a cancer patient using NGS to receive personalised treatment, was used in all interviews to facilitate the discussion process and provide a point of continuity across interviews.

With participants’ consent, interviews were recorded and transcribed into both Greek and English. Transcripts were analysed using thematic analysis as described by Braun and Clarke (2006). Initial codes were generated, and then, themes were identified, defined and named. An initial coding frame was generated from the research questions which acted to guide, but not constrain, the analysis. Interviews were coded using NVivo, and themes and sub-themes were developed and iteratively revised.

Three clinicians, two experts with bioethical background and five geneticists, four of whom also wore the “hat” of a genetic counsellor, were interviewed. Given the small number of professionals working in this area in Greece, we have chosen not to give job titles and/or roles when presenting the results below due to the risk of unintentionally revealing participants’ identities. Instead, we use simple numbers to tag each quotation.

## Results

### Why IFs from clinical sequencing are challenging

Our experts considered that NGS should be considered as “the last resort” and should therefore be ordered only when all other tests have failed to give a diagnosis. Clinicians especially reported that they are trying not to generate IFs by selecting targeted sequencing rather than NGS and by keeping NGS as the “last resort”. Clinicians believed that using NGS in the clinical setting would create problems because “if you start looking, you will definitely find something”. Therefore, for the time being, targeted sequencing would be more useful.

*For me it is rather simple. If symptoms resemble Huntington*’*s for example I will order a test only for that. I won*’*t start looking around. I won*’*t even use genetic testing unless I have to. I am not saying that it is not useful, because it is, and occasionally we have managed to diagnose conditions that we couldn*’*t have done otherwise*, *but if I can use other kinds of testing I would rather do that. With genetic testing you never know what you will get* (*Participant 10*).

*Not even for cancer. If later we discover that all cancers are hereditary maybe then but until then I would only use genomic testing rarely in extreme cases* (*Participant 04*).

Although Greek experts noted that there are some similarities with other areas of medical practice that can provide a starting point, clinicians reported that the concept of IFs is well integrated in the medical philosophy and they have been “taught” how to handle them during their medical training.

*But IFs are not something you could only have in genetic testing. We always knew that could happen* (*Participant 04*).

*Most tests could give you IFs. We have been trained and we always knew that the more you look the more you will find. It might be even more with genetic testing but the idea is the same* (*Participant 10*).

Additionally, they all reported having experience of handling IFs from other types of genetic testing and thought this would be of some help when dealing with IFs deriving from NGS testing.

*We have been thinking about this for a long time now. Especially with arrays* [*array*-*CGH* (*Comparative Genomic Hybridization*)] *we have found unexpected things more than once. It*’*s not something new* (*Participant 05*).

*Oh*, *yes. We are used to having IFs. We have them in prenatal testing very often. Ever since we started using the classical karyotype. You are looking for one thing and you find something else. Now we are going to use all this experience for clinical sequencing. This is not new to us* (*Participant 07*).

Previous experience from other types of testing could inform practices about IFs from clinical sequencing (e.g. IFs discovered during prenatal tests using cytogenetic tests); yet, experts considered that IFs differ in important ways. First, all participants reported that a very important difference was that genetic information affects more than just the actual patient or the person getting tested. The nature of genetic information makes it unique and complex because it is shared by all family members, even those not affected by the genetic condition in question.

*What is different this time is that family members have even a legal right to have access to that information. Because it could affect them too* (*Participant 01*).

*Learning any genetic information is something you should share. It doesn*’*t affect only you. People need to overcome their spontaneous reaction of hiding something that is bad and share it. This might make a difference in other people*’*s lives. They might have the opportunity to get tested*, *follow up even have a treatment. It is a moral obligation* (*Participant 03*).

A second important factor that was acknowledged by most participants was that this is an area in which knowledge and scientific understanding is constantly developing. This needs to be taken into account when making choices about the results that should be returned.

*The problem with genetics is that we think we know something today and then in a year*’*s time it is proven wrong or insufficient. We can*’*t pretend we know everything because we don*’*t* (*Participant 02*).

*Because everything changes so quickly we might have to consider keeping findings and returning them on a later time if we are not sure what they mean now* (*Participant 05*).

Third, there was a consensus among all experts that when using clinical sequencing, especially NGS, it is the interpretation of the results that is important, not the test itself.

*Anyone could buy the equipment for NGS but there are only a few who could interpret results. And there is the whole importance. Because we will get so many results*, *we will have a look and using specific software we will throw 1998 or 1999 out of 2000. The remaining ones we will see. We will have to think about them and consider the family as well* (*Participant 08*).

Fourth, clinicians in particular also suggested that genetic conditions differ in another important way: most genetic conditions are not actionable. For some conditions the only “action” that could be taken would be the option of prenatal or preimplantation diagnosis, if available, as no preventive measures were available.

*The problem is that for most genetic conditions there is nothing you can do*! *Only be informed*, *follow-up and help other make reproductive choices if you can* (*Participant 04*).

*A patient with a hereditary genetic condition comes very close to his doctor. It*’*s not like having a respiratory condition that he could take two sprays* [*respiratory drug*] *and get well. Here you have many issues*, *social*, *psychological*, *moral* (*Participant 10*).

Fifth, returning genetic information to patients differs from returning other health-related information because learning genetic information has the potential to change someone’s life, especially if it is unexpected and serious. Many participants suggested that when conveying “bad news”, the support of a clinical psychologist would be vital.

*Especially if what you are going to tell them is really bad you need there a psychologist. They will know better how to help them* (*Participant 05*).

*We had a psychologist at some point as a member of our group when disclosing such information. And that made a great difference. Because these people need support in more than one way and long*-*term support* (*Participant 10*).

Finally, particularly in the Greek context, genetic information might have another special characteristic. Participants stated that Greek society remains relatively traditional in certain domains. The experts interviewed suggested that being diagnosed with a genetic condition could lead to stigmatisation. This could discourage families, especially parents, from disclosing a genetic diagnosis even to their children. In this way, children are being deprived of the opportunity to follow up and make relevant reproductive choices.

*We are having mothers of teenagers or young adults coming here and they say* “… *how would we manage to find her a husband if people would know that we have that*?” *and they don*’*t tell them anything. And then their kids grow up and have kids of their own and they don*’*t have the chance to use prenatal or pre*-*implantation diagnosis and they end up having kids with serious juvenile form of these conditions and when they learn that they could have known and could have done something about it they so disappointed. They would do everything to avoid being stigmatised. We face that very often here* [*in Greece*] (*Participant 10*).

### How IFs are currently returned

Regardless of the concerns expressed, clinicians order less targeted sequencing and IFs are being generated. Currently, when IFs are discovered, they are managed at a “local” level, i.e. within the clinic or the laboratory, on an ad hoc basis. Clinicians and geneticists reported that they meet together and discuss cases as they arise. Results, including any IFs, are then discussed between the ordering clinician, a geneticist and a genetic counsellor (if there is one available), or a team consisting of clinicians and geneticists.

*For the time being we are working all together. Clinicians bring the geneticists and with help from the social service of the hospital we make a decision. The social service has helped us quite a lot. But not all hospitals have one*! (*Participant 10*)

*If something like that would happen the only thing we can do is to discuss it all together, there is nothing else* (*Participant 03*).

All results, both diagnosis-related and IFs, are given to patients during a genetic counselling session where the clinician or th*e* geneticist is acting as a genetic counsellor. The results are being returned orally and also in writing.

*Here we are also acting as genetic counsellors as well. There is no one else to disclose results. Physicians neither can nor want to do it. They know they are not trained for it*, *and neither are we but since there is no-one else*, *we have to* (*Participant 08*).

*We give results during genetic counselling but we also hand them a report to have it for their personal medical record* (*Participant 03*).

Although this was the current practice reported, experts expressed differing views on who should return results. All experts underscored the importance of having a person who is properly trained for this task but their definitions of “properly” differed. Clinicians believed that they were the most appropriate group, while geneticists and experts with a bioethical background thought that results should be disclosed by a multidisciplinary team. This team should consist of not only clinicians but also other professionals, such as geneticists and clinicians specialised in the relevant condition (e.g. oncologist if a cancer susceptibility gene had been discovered). At the same time, most of the experts questioned the appropriateness of clinicians not specialised in genetics dealing with genetic tests and the results, especially when NGS is used. They were of the opinion that non-specialist clinicians lacked the expertise to explain the procedures and to provide pre- and post-testing counselling. The lack of a recognised specialty of “clinical geneticist” made things even harder.

*To understand that*, *here we are acting as genetic counsellors because we don*’*t have genetic counsellors and doctors don*’*t know what to do. They are asking for our help and sometimes even we don*’*t know what to do* (*Participant 05*).

*Not to mention that we don*’*t even have a specialty recognised*! *(Participant 02*)

### Which results should be returned?

Most experts mentioned the concept of “patient autonomy” and understood this as each patient’s individual right to choose whether or not to be told about IFs, although their ideas about the best way to achieve this varied.

*We need to make sure that they are informed well enough and that they are deciding autonomously. We should give them all the information we can and let them decide by themselves* (*Participant 03*).

*Whoever is doing the genetic counselling should provide all the available information. They should let them know that IFs could be discovered. And then it is on the individual*’*s responsibility to ask his doctor if they indeed discovered something. This way we would be sure that the individual actually wants to learn the findings. If it is the doctor that asks then that is not exactly autonomous*! *They need to actively participate*! (*Participant 01*)

However, it seems current practice is not always guided by this principle. Clinicians admitted they do occasionally adopt a more paternalistic approach and try to act in what they think is their patient’s best interest, even if this means making some preliminary decisions by themselves.

*Even if the patient has asked for all results we won*’*t give him everything. We will definitely give him clinically valid and clinically actionable ones*, *or results that concern serious of life-threatening conditions but about the rest of them* … *I don’t know. We will discuss about it and according to what we will decide we will let him know* (*Participant 06*).

*We won*’*t give him everything. We will discuss it and we will decide what he needs to know* (*Participant 08*).

Importantly, most experts believed that patients and their families, who may be asking for all available information to be returned, are not in a position to deal with genetic results and will have even greater difficulty understanding results that are unexpected and unrelated to the original diagnosis.

*They* [*patients*] *want to know as much information as they can. Few are those saying that they don*’*t want to know. If they could afford it they would want to do every kind of test they could*! *But they have a hard time when you actually get back at them with results. They don*’*t know what to do with it*, *especially with multi-factorial conditions* (*Participant 06*).

*In Greece yes*! *They want to know everything. They ask for everything. And they want us to test them for all available genes*.

(*Interviewer*: *And do you think they are handling these results*?)

*No*, *no way. They definitely cannot*! *They don*’*t really know what they ask for* (*Participant 04*)

Experts believed that the only way to support these families was by spending a considerable amount of time with them giving pre-testing counselling where they try to explain everything according to the patient’s needs and level of understanding.

*How much they* [*patients*] *can understand is related to how much time you spend with them and how patient you are. According to the literature we are supposed to have a one-and-a half-hour counselling session. And we are doing that here. Our slogan is that you won’t leave unless you understand*! (*Participant 10*)

Therefore, notwithstanding their awareness of the patient’s right to choose, all participants had their own ideas about which results should be returned and when. All believed that clinically valid and actionable results should be returned. Interestingly, not all of them seemed to think about “actionability” in the same way. Some saw actionable as meaning only results that could lead to treatment, while others also included results that could provide other family members with the opportunity to make different reproductive choices even if no intervention was available.

*Only if there is a treatment available. If there is none then what’s the point of telling them*? (*Participant 01*)

*If there is something they could do about it then yes*. […] *if they want to have a child they should know to be able to use prenatal or preimplantation testing to try to avoid that condition* (*Participant 04*).

Regarding returning IFs to minors, experts stated that results should be returned in cases where there could be an impact on patients’ reproductive choices or when there would be an opportunity to follow up or have access to preventive measures for minors in the future. Several experts expressed their concern regarding IFs about late-onset conditions, believing that such findings could cause more harm than good. Clinicians were slightly less willing to disclose results compared to geneticists.

*Let*’*s say you find Huntington*’*s in a 5-year old boy*, *that is a finding you can*’*t neglect. But what is the point of returning a result about something that will happen 40 years later*? *There is a huge one*! *Science is evolving so quickly and that kid could be able to do something to delay the symptoms or participate in a clinical trial much sooner* (*Participant 10*).

*For me to inform someone for something that will happen 20–30 years later doesn*’*t make sense. You force him to* “*medicalise*” *his life. I don*’*t think he needs to know. Not for something that will happen that far away. Especially if there is nothing he can do about it. He could learn about it later. I prefer to inform them for something that will happen in the near future* (*Participant 01*).

There were differing opinions about results that are clinically valid but not clinically actionable. Clinicians were less willing to return them than geneticists or professionals with a bioethical background, but they did all agree that they would like to know their patient’s wishes in advance. As above, the importance of pre- and post-testing counselling was underlined by all experts in these cases and all agreed that if a patient had consented to receive results, then, his or her wishes should be respected.

### What needs to change in Greece?

As discussed earlier, currently, there is no framework to guide practice in Greece. All experts noted the lack of any legal documents, guidelines or other supportive mechanism to support clinicians, geneticists or the laboratories using sequencing technologies if IFs are discovered.

*There is nothing. Absolutely nothing! No supportive mechanism*, *no laws. Nothing*! *Every laboratory has*, *in best case scenario*, *done what we have done. We have an ad hoc process to solve problems like that. We all meet* [*clinicians*, *geneticists*] *and discuss case by case* (*Participant 04*).

Many experts expressed their disappointment about the current situation in Greece and their belief that things would not change easily. Two key things are needed, according to those interviewed: better public understanding and clear guidelines to support professionals.

*Lay people should be educated about genetics. Because in Greece we have many genetic conditions. In certain areas because of inbreeding the prevalence of genetic conditions is huge. People should learn about it. And they should also learn about the nature of genetic information. And we need studies reporting the frequency of genetic conditions in Greece* (*Participant 10*).

*We should have a consensus among stakeholders*, *clinicians*, *professionals*’ *associations*, *geneticists. And all of them should describe a process*, *step-by-step the counselling process*, *something like guidelines and a leaflet that could be distributed to lay people before using clinical sequencing* (*Participant 07*).

When asked if they would like to have a list of conditions for which IFs should be returned, such as the list prepared by ACMG in the USA, the majority stated that because a list could never be complete, it would be better to have guidelines describing the criteria, rather than the conditions, for which IFs should be returned.

*We need a committee to prepare a catalogue*, *a list with all the necessary rules. You can*’*t describe all conditions* (*Participant 05*).

*We need a list with the criteria not a list of genetic conditions. Guidelines for all laboratories describing what results should be returned*, *in what age*, *the severity of condition*, *what would happen with late-onset*, *with minors* …*things like that* (*Participant 06*).

Finally, many suggested that we do not need to “re-invent the wheel” but we could instead look to what was available in other countries and adapt it to the Greek context.

*I would like to have some short of soft-law*, i.e. *guidelines from a professional association that would describe what is happening in other countries*, *what is the state of the art abroad. And from that they could bring something and adapt it according to our need here. We don’t need to start from the beginning when there could be something available abroad* (*Participant 09*).

## Discussion

Our goal was to investigate Greek experts’ attitudes toward clinical sequencing and return of IFs. Their extensive experience and expertise was used to help us acquire a better understanding of the existing situation in Greece regarding clinical sequencing and the return of IFs.

From the interviews, a consensus could be observed among experts from different backgrounds that IFs that are clinically valid and actionable should be returned, always according to patients’ wishes. In the same way, they all acknowledged the importance of pre- and post-test counselling and the fact that when it comes to NGS testing, interpretation of results is the area requiring the most attention. Most experts agreed that IFs discovered in minors should be returned in most of the cases but with extra caution. Finally, they all insisted on the need to have guidelines as soon as possible but preferred a list with criteria and detailed counselling advice rather than simply a list of genetic conditions they would be required to search for and if found, about which they would need to inform their patients.

On the other hand, no consensus could be found regarding what actions should be taken regarding clinically valid but non-actionable results and the best time to return IFs. Several differences were observed between clinicians and geneticists. Clinicians preferred more targeted genetic testing while geneticists were more willing to use NGS. Additionally, clinicians were less in favour of returning non-actionable results and informing a patient’s family of them.

Greek experts seemed to consider that genetic testing, and the genetic information derived from it, differs in some important ways from other medical information, as this data concerns family members apart from the patient and scientific knowledge and understanding change very quickly in this context. Additionally, the meaning of actionability was also raised by many and understood in more than one way. Patient autonomy was referred to as an ideal, but problems with managing this in practice were highlighted.

Our findings are in agreement with studies conducted elsewhere, both from clinical settings and research, and suggest that a consensus exists only regarding IFs that are both clinically valid and actionable (Downing et  al. 2013; Facio et  al. 2011, 2013; Lohn et  al. 2013; Brandt et  al. 2013; Green et  al. 2012; Lemke et  al. 2012; Townsend et  al. 2012; Dimmock 2012). Theoretical and more philosophical approaches have also suggested that, at least for the time being, only these should be disclosed (Berg et  al. 2011; Goddard et  al. 2013; McGuire et  al. 2008). The same is true for results from genetic research in general (Abdul-Karim et  al. 2013), research using NGS (Klitzman et  al. 2013) or research involving biobanks (Goldman et  al. 2008; Meulenkamp et  al. 2012).

The importance of pre- and post-test counselling and the need to provide individual support depending on patients’ needs and understandings was also mentioned. As suggested elsewhere (Middleton et  al. 2007), depending on their needs, patients develop different relationships with their clinicians or genetic counsellors so the patient’s preferences should be taken into consideration. The use of NGS would require very long counselling sessions, over 5 h, making it impractical and with questionable utility for patients (Ormond et  al. 2010). As our experts suggested, spending time with patients would make a difference; it might be worth considering that alternatives are needed to support patients with other ways apart from prolonging the counselling session. Finding the right balance between providing enough information to help a patient to make an informed decision and providing too information that it becomes “counterproductive” (Ormond et  al. 2010) is another challenge that needs to be faced before the full integration of NGS in the clinical setting.

Greek experts seemed particularly concerned about potential stigmatisation, noting that Greek society might be more traditional than others and individuals might feel discouraged to disclose genetic information even within the family. Although potential discrimination and stigmatisation have been discussed in other studies about receiving results from clinical sequencing (Downing et  al. 2013; Townsend et  al. 2012), or participating in research (Halverson and Ross 2012), concerns about disclosure within a family are rarely mentioned (Clarke et  al. 2005; Wilson et  al. 2004). Our clinicians suggested that parents might not feed back results to their children or anyone else in their family, because they are afraid that their offspring might have difficulties in getting married if associated with a diagnosed genetic condition. This finding is also discussed among BRCA carriers (Dimillo et  al. 2013) or patients with neurodegenerative diseases (Paulsen et  al. 2013). Usually, stigmatisation and potential discrimination are discussed in relation to mental health conditions (Yang et  al. 2013) or in regard to health insurance (Kass et  al. 2007) but has not actively been discussed in the context of IFs.

The Greek experts interviewed would prefer to decide which results to feed back according to their clinical discretion, and they stated that, for the time being, this should be done on a case-by-case basis. They would prefer not to have a list of conditions for which they would be required to report, but a list of criteria to help clinical decision-making and prioritising results. Additionally, clinicians in our sample clearly expressed a preference toward more targeted tests to avoid the discovery of unrelated findings that would be difficult to feed back and might be confusing and disorienting for patients. As other commentators have suggested “[A]n informed, targeted approach to genome analysis makes the clinical test a more discrete and definable entity that is possible to interpret and reduces unwanted incidental findings” (Wright et  al. 2013, p. 3). Greek experts seemed to understand a patient’s autonomy in different ways and, as has been suggested elsewhere (Ross et  al. 2013; Klitzman et  al. 2013).

Regarding the disclosure of IFs directly to family members, not through the patient, our experts seemed to be willing to proceed with caution, especially when IFs were serious. This “duty to warn family members of inherited health risk” (Offit et  al. 2004) has been discussed elsewhere and health-care professionals have suggested that they have a responsibility to encourage but “not to coerce the sharing of genetic information in families” (Storm et  al. 2008). However, failure to warn family members about hereditary disease risks has already resulted in three lawsuits against physicians in the USA (Offit et  al. 2004) while recent changes in Australian law now allow disclosure to relatives (Otlowski 2013). These changes suggest that this issue requires further research in order to assist clinicians. Legal and professional responsibilities should be clarified to avoid driving clinicians to over or under investigate and report because of fear of repercussions (Wright et  al. 2013).

## Conclusion

Experts from Greece reported the lack of any supportive mechanisms, even though clinical sequencing is integrated in the health services available to patients. The availability and use of sequencing in the clinical setting is expected to increase, and experts are asking for guidelines to support them with the return of clinically valid and actionable results. Further research in Greece is needed to seek the exact type of guidelines that should be created as well as to investigate cultural differences between nationalities and cultural and professional groups in Europe and internationally.

Although our results should be treated with caution due to the small sample size, we believe we have demonstrated the current situation regarding clinical sequencing in Greece. The preparation of guidelines for Greece could follow examples set in other countries, but there is a clear need to ensure that they reflect the Greek situation. Country-specific characteristics should be taken into consideration such as cultural lay beliefs and a health-care system that is mainly based on professionals’ discretion and willingness to act on patients’ best interest, despite the lack of any clear support from the system. Such guidance will allow experts in Greece to continue to provide excellent and thoughtful care for their patients.
